# The Classification of Aneurisms

**Published:** 1895-06-08

**Authors:** A. Pearce Gould

**Affiliations:** Senior Assistant Surgeon to the Middlesex Hospital


### The Classification of Aneurisms.

By A. Pearce Gould, F.R.C.S., Senior Assistant Surgeon to the Middlesex Hospital.

It is a strange fact that, although the whole subject of aneurism has excited great interest in all surgeons, especially since Hunter's historic operations, there has yet been a good deal of divergence in the various classifications of aneurism that have been drawn up. And the classification of these tumours is really a very important matter, particularly as terms have been employed that must unconsciously influence the thought of those who use them. Various methods of grouping aneurisms may be employed; thus they may be classified according to their shape, into tubular, fusiform, and globular or sacculated; or according to their contents whether solid or fluid; or according to their clinical course, whether stationary, enlarging or contracting. But all such grouping is a little beside the mark. For what is the most important point that we ought to express in our classification? Surely the most important fact is whether or not the wall of the aneurism is a complete sac, and strong enough to resist the pressure of the blood upon its interior. When that is the case the danger of the disease is vastly less than where the sac is imperfect or weak, and unable to withstand the force of the circulation. The great factor in the importance of any aneurism is the degree in which the expansile force of the circulation is balanced by the resistance of the sac. If the balance is true, the tumour remains small, is stationary, or grows very slowly, and the surrounding parts, however important they may be, have time to accommodate themselves to the pressure;—the symptoms in such a case are few. But when the balance is untrue, the sac is expanded more or less rapidly, the tumour therefore grows rapidly, may even attain a great size, and the pressure upon the surrounding parts is severe;—the symptoms are very grave. Still more when the sac is incomplete, and allows the blood to escape into the tissues around it, do the effects become aggravated, for we have to deal with a subcutaneous breach in an artery;—the symptoms become extremely urgent.

There are no tissues in the body so well adapted to resist the pressure of the blood as those composing the healthy wall of an artery. What we are apt to regard as "stronger" tissues are not so well endowed in this respect. Bone exposed to this force is rapidly absorbed, and tough, strong aponeuroses and fasciæ yield under it, the fibres are separated, and they form no efficient barrier to the blood. It is the wonderful muscular contractility and elasticity of the arteries that fits them for their special function. The further the wall of an aneurism departs in structure from a healthy arterial wall the less able is it to withstand the force of the heart, and to form an efficient barrier to the escape of the blood from it.

There is one form of aneurism in which the sac is formed of all the coats of the artery, and in which, therefore, growth is slow and the danger of rupture very small. It is that form in which from a general degenerative change in the vessel its elasticity is diminished and its whole circumference yields. It is known as a tubular or fusiform aneurism, these names having been given to it from its usual form, but it is not the form of the tumour so much as the nature of the sac that really distinguishes it from others. If at any spot in a fusiform aneurism the arterial walls are more greatly weakened by disease, a local bulging occurs, and the sac of this pouch being thinned and weak yields more rapidly and is liable to rupture. This is a not infrequent event, and such cases are well designated mixed aneurisms.

When an aneurism originates in the yielding of a diseased spot in the wall of an artery, the sac is formed by less than all the coats of the vessel, it is from the first too weak to resist the expanding force of the circulation; it therefore grows fast and is liable to burst. Such an aneurism is known as a sacculated aneurism; the name appears to have been given on account of the general form of the tumour, its appearance as a sac springing from one side of the vessel; globular aneurism would be as good a name, for the possession of a "sac" is not its distinguishing feature, but rather the want of a really efficient sac. It may happen that at its very earliest formation the sac of such an aneurism may consist of all the coats of the artery. Such an event is certainly quite rare, if it occurs at all, and very quickly the more diseased inner coats of the vessel disappear, and the only part of the arterial wall to be found in the sac is the stretched outer coat with, perhaps, a few scattered bundles of muscular tissue from the middle tunic. An attempt has been made to distinguish these two forms of the disease by the names "True" and "False," but as applied to sacculated aneurisms such names are inappropriate. An aneurism that has not all the coats of the artery in its sac is in no ordinary sense of the word less of an aneurism, less truly or typically an aneurism, than one in which all the coats are still present. Another objection to these terms is that it is quite impossible to distinguish between the two forms of sac by any symptoms during life, or, indeed, by any other means than very careful microscopical examination. "True" sacculated aneurisms are pathological curiosities, and the essential and most important feature of a sacculated aneurism is the absence from the sac of part of the arterial wall, at least. But if we reject this sub-division, we must be careful to make another and very vital one, depending upon the integrity of the sac. So long as the sac is entire the blood is all confined within it, but if the sac ruptures the blood escapes into the tissues beyond and around it, and this gives rise to the most urgent and even desperate symptoms. As the breach in the sac may be either a very small one, allowing only of a leaking of the blood through it, or a large rent, it is well to distinguish these two forms. Hence, sacculated aneurisms should be classified into "circumscribed"—meaning those with an entire sac, "leaking," and "ruptured."

